# Vascular refilling coefficient is not a good marker of whole-body capillary hydraulic conductivity in hemodialysis patients: insights from a simulation study

**DOI:** 10.1038/s41598-022-16826-8

**Published:** 2022-09-10

**Authors:** Leszek Pstras, Jacek Waniewski, Bengt Lindholm

**Affiliations:** 1 Nalecz Institute of Biocybernetics and Biomedical Engineering, Polish Academy of Sciences, Warsaw, Poland; 2 Division of Renal Medicine and Baxter Novum, Department of Clinical Science, Intervention and Technology, Karolinska Institute, Stockholm, Sweden

**Keywords:** Circulation, Haemodialysis

## Abstract

Refilling of the vascular space through absorption of interstitial fluid by micro vessels is a crucial mechanism for maintaining hemodynamic stability during hemodialysis (HD) and allowing excess fluid to be removed from body tissues. The rate of vascular refilling depends on the imbalance between the Starling forces acting across the capillary walls as well as on their hydraulic conductivity and total surface area. Various approaches have been proposed to assess the vascular refilling process during HD, including the so-called refilling coefficient (Kr) that describes the rate of vascular refilling per changes in plasma oncotic pressure, assuming that other Starling forces and the flow of lymph remain constant during HD. Several studies have shown that Kr decreases exponentially during HD, which was attributed to a dialysis-induced decrease in the whole-body capillary hydraulic conductivity (L_p_S). Here, we employ a lumped-parameter mathematical model of the cardiovascular system and water and solute transport between the main body fluid compartments to assess the impact of all Starling forces and the flow of lymph on vascular refilling during HD in order to explain the reasons behind the observed intradialytic decrease in Kr. We simulated several HD sessions in a virtual patient with different blood priming procedures, ultrafiltration rates, session durations, and constant or variable levels of L_p_S. We show that the intradialytic decrease in Kr is not associated with a possible reduction of L_p_S but results from the inherent assumption that plasma oncotic pressure is the only variable Starling force during HD, whereas in fact other Starling forces, in particular the oncotic pressure of the interstitial fluid, have an important impact on the transcapillary fluid exchange during HD. We conclude that Kr is not a good marker of L_p_S and should not be used to guide fluid removal during HD or to assess the fluid status of dialysis patients.

## Introduction

During typical hemodialysis (HD), the microvascular absorption of fluid from tissues combined with the lymphatic flow (i.e. the partial reabsorption of the afferent lymph in the lymph nodes and the venous drainage of the efferent lymph^1^) constitute the so-called vascular refilling that compensates for the reduction of blood volume due to ultrafiltration in the dialyzer^2–4^. The refilling of the vascular space during HD (or plasma refilling, as some call it^5^) is a crucial process not only for keeping the blood volume relatively stable or only slightly reduced, thus ensuring hemodynamic stability, but also for enabling the efficient removal of the excess body fluid that is present predominantly in the interstitial space of body tissues.

The rate of microvascular fluid exchange (J_v_) depends on the imbalance between the so-called Starling forces acting across the semi-permeable capillary walls (and to some extent post-capillary venules) that include hydraulic/hydrostatic pressures and colloid osmotic (oncotic) pressures of blood plasma and interstitial fluid^6–8^ and is usually described using the following equation:

1 $$J_{v} = K_{f} \left[ {\left( {P_{c} - P_{is} } \right) - \sigma \left( {\pi_{pl} - \pi_{is} } \right)} \right]$$

where K_f_ is the capillary filtration coefficient equal to the product of capillary hydraulic conductivity/permeability per unit area (L_p_) and the total surface area of exchange (S), P_c_ is the capillary blood pressure (the sum of hydraulic pressure of the flowing blood and the hydrostatic pressure exerted by the weight of the blood column above), P_is_ is the hydrostatic pressure of the interstitial fluid, σ is the protein reflection coefficient^9^, and π_pl_ and π_is_ are oncotic pressures of blood plasma and interstitial fluid, respectively.

Under normal conditions, in the vast majority of tissues there is a net filtration of fluid out of capillaries^10^, which usually reverses into absorption of fluid from tissues during HD^2^. Assuming that Eq. (1) can be used to describe the transcapillary transport processes on the whole-body level (across the aggregated capillary wall, representative of the average body tissue), and that the total volume of erythrocytes remains constant, the changes in plasma volume (V_p_) during HD can be described as follows:

2 $$\frac{{dV_{p} }}{dt} = - J_{v} (t) + L(t) - UF(t) = {\text{R}} (t) - UF(t)$$

where L(t) is the rate of lymph drainage to the vascular system, UF(t) is the rate of ultrafiltration in the dialyzer (assumed constant), and R(t) is the total rate of vascular refilling.

For any non-zero net transcapillary pressure gradient (hydrostatic and/or osmotic), the higher the K_f_ (or L_p_S), the more fluid can pass across the capillary walls through various gaps or clefts between the endothelial cells, as well as via intracellular channels. Both hydraulic conductivity (L_p_) and capillary surface area (S) can, however, change under various circumstances, meaning that the global L_p_S is not necessarily constant.

In 1996, Tabei et al. proposed an index of vascular (plasma) refilling efficiency during HD (Kr)^11,12^ that describes the rate of vascular refilling per unit of oncotic pressure change and is defined as follows:

3 $$Kr(t) = \frac{{{\text{R}} (t)}}{{\pi_{pl} (t) - \pi_{pl} (0)}} = \frac{{\frac{{dV_{p} }}{dt} + UF}}{{\pi_{pl} (t) - \pi_{pl} (0)}}$$

They assumed (see the Supplementary material) that during HD, π_pl_ is effectively the only Starling force that changes and (inexplicitly) that the lymph flow is constant and σ = 1, and hence they proposed Kr as a substitute of L_p_S that can be estimated from the above equation by tracking plasma volume changes during HD and measuring the concentration of total plasma protein (to calculate plasma oncotic pressure, e.g. using the well-known equation by Landis and Pappenheimer^13^).

Tabei et al. showed that Kr decreases exponentially during HD, which they attributed mainly to a progressing reduction of L_p_S, likely due to the decreasing amount of plasma atrial natriuretic peptide (ANP) following its removal in the dialyzer^11,12^. It is, however, to be expected that during HD, due to ultrafiltration and intense microvascular fluid and solute exchange, Starling forces other than plasma oncotic pressure also change, just like the rate of lymph absorption changes with decreasing interstitial fluid pressure (not considered by Tabei et al.), and hence there may be another explanation for the observed decrease in Kr.

Therefore, the aim of this study was to analyze the relationship between Kr and L_p_S using a mathematical model of HD treatment in which all Starling forces as well as the flow of lymph are variables. In particular, we wanted to analyze the magnitude of intradialytic changes in all those variables in order to verify whether a decrease in Kr observed in dialysis patients results, indeed, from a dialysis-induced decrease in L_p_S or if it is caused by other mechanisms. We also wanted to analyze whether Kr (calculated at some point during HD) could be a good marker of LpS to be used for the assessment of patient’s fluid status or estimation of the dry weight, as has been proposed in the past^14,15^.

Note that Eq. (1) reflects the original Starling hypothesis of microvascular fluid exchange^6^ and hence represents the classic Starling principle^16^. This principle has been recently revised^10,17^ or rather extended^17^ to account for the effect of the glycocalyx layer (a relatively thin fibrous meshwork of proteoglycans and glycoproteins present on the luminal side of the endothelium^18^), which is believed to be the main barrier for the transcapillary transport of macromolecules^19–21^. According to the Michel-Weinbaum model^16,22,23^, behind this layer, in the clefts between the endothelial cells, there is a space that is ‘insulated’ from the bulk interstitial fluid by the tight junction strand with a limited amount of pore-like openings, so that the oncotic and hydrostatic/hydraulic pressure behind the glycocalyx are different than in the interstitial fluid, and hence for the calculation of microvascular fluid exchange one should use the trans-glycocalyx instead of trans-endothelial pressure gradients. In this study, for simplicity and to allow direct reference to the studies by Tabei et al., we employed the classic Starling principle and we discussed our approach in the “Discussion” section.

## Methods

### Mathematical model

The simulations presented in this study are based on our previously developed lumped-parameter model of the cardiovascular system with baroreflex integrated with the model of whole-body water and solute transport during HD implemented as a set of ordinary differential equations in MATLAB (The MathWorks Inc.). The model has been fully described in our previous work^24,25^, and hence here we present only a short overview (in particular, all equations and parameters of the three-pore model employed to describe the transcapillary transport of water and solutes^26,27^ are presented in our previous paper and the associated supplementary material^25^). The model has been developed for a comprehensive analysis of cardiovascular system, blood volume regulation, and whole-body solute kinetics during HD, and hence it includes features that are not necessarily needed for the study of microvascular fluid exchange. However, given that here we simulate a dialysis session in the assumed virtual patient with all model parameters fixed and not fitted to some data, having those features does not constitute an obstacle for the present study, while providing a more physiologically-based (albeit still simplified) description of the cardiovascular system that allows for a somewhat more accurate representation of changes in the capillary plasma oncotic pressure and capillary blood pressure, which would not be possible if we had only one plasma compartment or if we did not model the changes in venous pressure that are transmitted back to the capillaries. For these reasons, we decided to use the entire model in the previously developed form.

**Figure 1:**
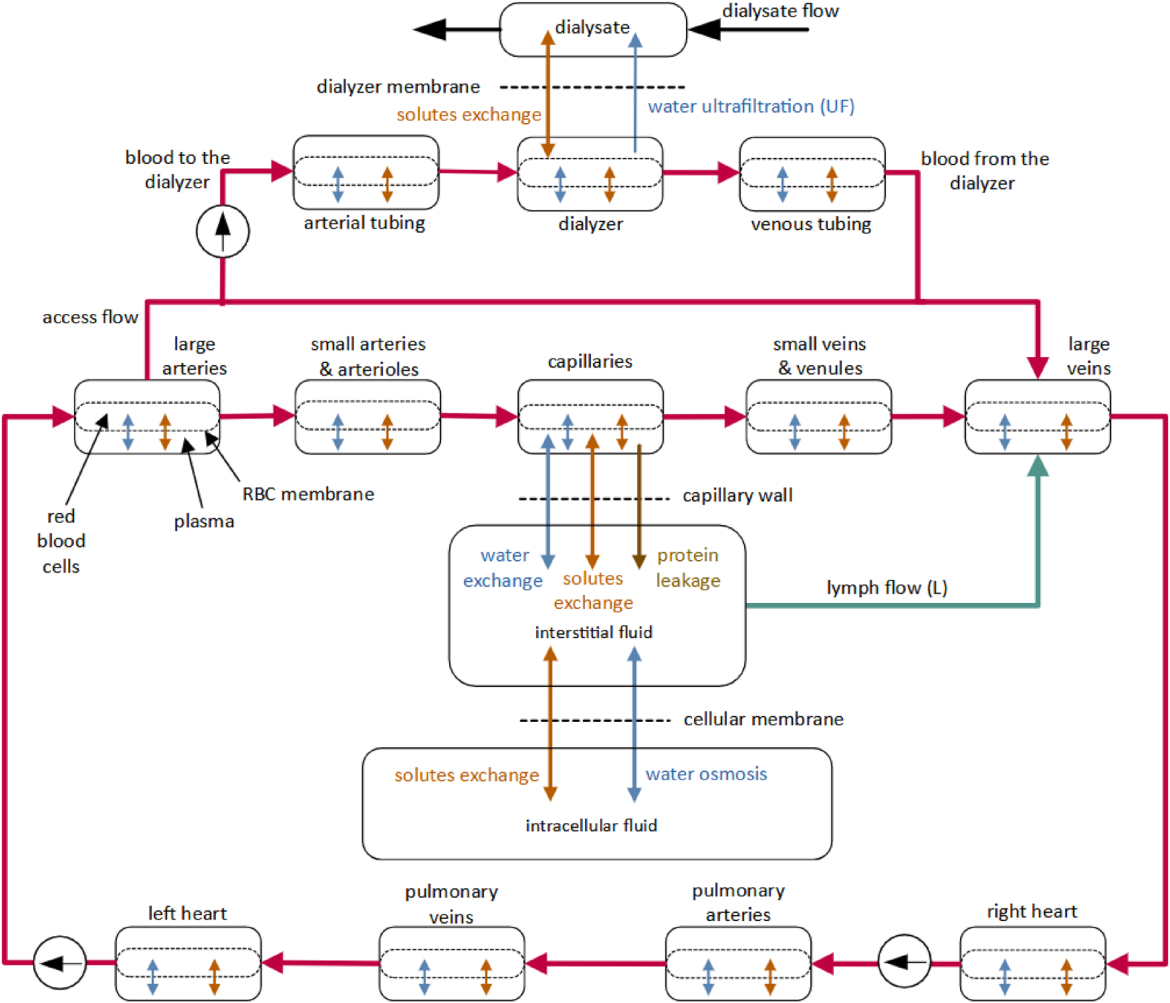
Overview of the compartmental model of the cardiovascular system and whole-body water and solute transport during haemodialysis^24^.

The cardiovascular part of the model includes nine blood compartments (large arteries, small arteries and arterioles, systemic capillaries, small veins and venules, large veins, right heart, pulmonary arteries, pulmonary veins, and left heart) and three extracorporeal compartments (connected via arteriovenous access) with the two-phase blood flow (plasma and erythrocytes) driven by two continuous-flow pumps representing the pumping action of heart ventricles (see Fig. 1). The baroreflex includes four mechanisms controlling heart rate and contractility (affecting the pump outflow), resistance of small arteries and arterioles, and the unstressed volume of small veins (venous capacitance) based on pressure signals from low-pressure area (right heart compartment) and high-pressure area (large arteries compartment).

The microvascular water and solute exchange was modelled using the well-established three-pore model of the capillary wall^26,27^ with three pathways for solute and/or water transport reflecting the structural properties of continuous (non-fenestrated) capillaries: (1) small pores representing the inter-endothelial clefts covered by the glycocalyx layer^10,21^, (2) large pores representing large gaps in the endothelium and glycocalyx or vesicular transport of macromolecules^20,28^, and (3) ultrasmall pores representing aquaporins—a water-exclusive pathway^10^. We assumed that the large, small, and ultrasmall pores have radii of 250 Å, 45 Å, and 2 Å, respectively^26^, to which we attributed 5%, 85%, and 10% of the total (whole-body) capillary hydraulic conductivity, respectively^25^ (the latter has been assumed at 4.5 mL/min/mmHg^2,29^). For each pore type, the transcapillary fluid flow was modelled using an extended version of Eq. (1) to include different protein fractions (p) and to account for the possible osmotic pressure gradient due to small solutes (s)^24,25,30^:

4 $$J_{v,i} = \alpha_{i} K_{f} \left[ {\left( {P_{c} - P_{is} } \right) - \sum\limits_{p} {\sigma_{p,i} \left( {\pi_{pl,p} - \pi_{is,p} } \right)} - \sum\limits_{s} {\sigma_{s,i} \varphi_{s} \left( {c_{pl,s} - c_{is,s} } \right)RT} } \right]$$

where α_i_ is the fraction of K_f_ contributed by the i-th type of pore (Σ_i_ = 1), σ_p,i_ and σ_s,i_ are the capillary reflection coefficients for protein p and small solute s, π_pl,p_ and π_is,p_ are colloid osmotic (oncotic) pressures exerted by protein p in blood plasma and interstitial fluid, φ_s_ is the osmotic activity coefficient of solute s, c_pl,s_ and c_is,s_ are molar concentrations of solute s in the water of plasma and interstitial fluid (excluding the Gibbs-Donnan effect for small ions that is already included in the equations for π), and RT is a constant (= 19.3 mmHg/mmol/L).

We divided all plasma proteins into two fractions (albumin and non-albumin proteins treated collectively as globulins) and we estimated their contributions to the total oncotic pressure using the following equations^25^ (derived from Landis-Pappenheimer equations^13^ using the approach by Nitta et al.^31^ assuming the normal albumin-to-globulin ratio of 1.5):

5 $$\pi_{alb} = a\left( {2.8\,C + 0.18\,C^{2} + 0.012\,C^{3} } \right)$$

6 $$\pi_{glob} = \left( {1 - a} \right)\left( {1.1\,C + 0.13\,C^{2} + 0.005\,C^{3} } \right)$$

where C is the total protein concentration (in g/dL) in plasma or interstitial fluid, and a is the albumin mass fraction of total protein.

We assumed that the mean pressure of capillary blood (P_c_) is resistant to changes in arterial pressure (the auto-regulatory capacity of the capillary bed), whereas 80% of changes in venous pressure are transmitted to the capillaries^24^. P_c_ is therefore calculated as:

7 $$P_{c} = P_{c,0} + w_{v} \cdot (P_{sv} - P_{sv,0} )$$

where P_c,0_ is the initial capillary pressure calculated from the initial steady state, w_v_ is a parameter (0.8), and P_sv,0_ is the initial (normal) pressure in the small veins compartment (12 mmHg)^24^.

The hydrostatic pressure of the interstitial fluid was described as a linear function of the interstitial volume^24^:

8 $$P_{is} = P_{is,n} + \frac{1}{{C_{is} }} \cdot \left( {V_{is} - V_{is,n} } \right)$$

where P_is,n_ is the normal interstitial pressure (assumed at −1 mmHg^32^) corresponding to the normal interstitial volume (V_is,n_, assumed 15 L^24,33^) and C_is_ is the interstitial compliance, which was assumed to be 12% of normal interstitial volume per mm Hg^32^.

The lymph flow was described by linear functions of the interstitial pressure, as done by Gyenge et al.^24,30^.

9 $$Q_{L} = Q_{L,n} + LS \cdot \left( {P_{is} - P_{is,n} } \right),\,\,\,\,\,\,\,\,\,\,\,\,\,P_{is} \ge P_{is,n}$$

10 $$Q_{L} = Q_{L,n} \frac{{\left( {P_{is} - P_{is,ex} } \right)}}{{\left( {P_{is,n} - P_{is,ex} } \right)}},\,\,\,\,\,\,\,\,\,\,\,\,\,P_{is,n} \ge P_{is} \ge P_{is,ex}$$

where Q_L,n_ is the normal steady-state afferent lymph flow (assumed 8 L/day^8,33^) corresponding to the normal interstitial fluid pressure, P_is,n_, LS is the lymph flow sensitivity to interstitial pressure changes (assumed at 43.1 mL/mmHg/h^34^) and P_is,ex_ is the pressure of the interstitial fluid when its volume decreases to the volume excluded to proteins (V_is,ex_), at which point the lymph flow ceases (assumed at 50% of normal interstitial volume^32^).

For simplicity, we assumed that all afferent lymph is drained instantaneously to the large veins compartment, thus neglecting the partial absorption at lymph nodes (to the small veins compartment), which would not make any difference in our compartmental model.

The transcapillary protein transport was described as a combination of diffusion and convection through large and small pores^25^ (for equations and parameters, please see our earlier work^25^).

### Initial conditions

The model has been defined for pre-dialysis steady-state conditions in a virtual patient assuming 3L of fluid overload divided between the vascular space and interstitial fluid proportionally to their volumes in a healthy 70-kg man. Normal physiological pressures were assumed for all blood compartments. All model parameters are based on literature data^24,25^.

### Hemodialysis procedure

Unless stated otherwise, all simulations consider a standard 4-h HD session with 3L ultrafiltration. Two different priming procedures were considered^35^: 1) priming saline discarded when the extracorporeal circuit is being filled with the patient’s blood, and 2) priming saline infused to the patient. In both cases, we assumed the volume of the extracorporeal circuit of 220 mL (100 mL for the dialyzer and 120 mL for the tubing). For the case with the priming saline infused, the net ultrafiltration rate was increased accordingly to reach the same total net ultrafiltration. The dialyzer blood flow rate, solute clearances/dialysances, and the composition of the dialysis fluid were all the same as in our previous work^24,25^. In all simulations, t = 0 represents the start of HD (after the priming procedure).

### Simulation variants

In the basal simulations we considered a constant value of the whole-body L_p_S equal to 6 mL/min/mmHg^2^. We also performed HD simulations with lower (4 mL/min/mmHg) or higher L_p_S (8 mL/min/mmHg) as well as with L_p_S changing linearly from 6 mL/min/mmHg to 4 or 8 mL/min/mmHg during HD. Finally, we simulated HD for different levels of ultrafiltration (± 20%) in the same virtual patient (with the assumed 3L fluid overload) to analyze the impact of blood volume changes on Kr. For each case, Kr(t) was calculated from Eq. (3) using Eqs. (5) and (6) to estimate the total plasma oncotic pressure, with the total plasma protein concentration and the rate of plasma volume changes simulated by the model.

### Sensitivity analysis

The relative local sensitivity of Kr to each model parameter, θ_k_, was calculated as follows^36,37^:

11 $$S_{k} (t) = \left. {\frac{\partial Kr(t)\,}{{\partial \theta_{k} }} \cdot \frac{{\theta_{k} }}{Kr(t)}} \right|_{{\theta_{k} = \theta_{k,0} }} ,\quad \theta_{k} ,Kr(t) \ne 0$$

where the derivative was computed using the central difference approximation with the parameter θ_k_ increased and decreased by 0.01%.

The studied model parameters (166 in total) included the parameters describing: the cardiovascular and extravascular compartments (normal pressures and volumes, compliances, etc.), baroreflex mechanisms (gains, amplitudes, time constants), cardiac function, water and solute transport across the capillary walls and cellular membranes (permeabilities, reflection coefficients, etc.), dialysis session (ultrafiltration rate, blood flow rate, composition of the dialysis fluid, solute clearances/dialysances). The original values and context of all these parameters may be found in our previous work^24,25^.

## Results

**Figure 2:**
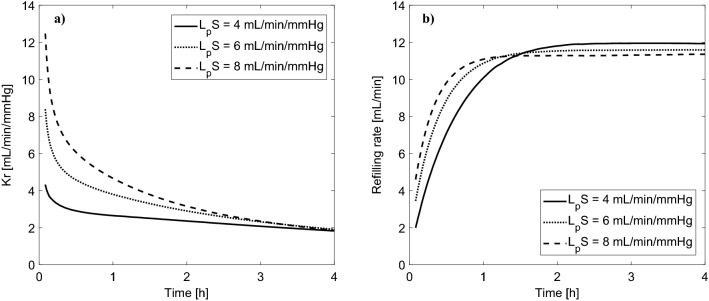
Simulated changes of (**a**) vascular refilling coefficient (Kr) and (**b**) vascular refilling rate (the sum of transcapillary fluid absorption and the total flow of lymph) during 4 h hemodialysis sessions with different values of the whole-body capillary hydraulic conductivity (L_p_S), with the priming saline discarded.

The simulated changes of Kr and the rate of vascular refilling (the sum of transcapillary fluid absorption and the total flow of lymph) during a standard 4 h HD session with the priming saline discarded are shown in Fig. 2 for different values of L_p_S. In all cases, Kr follows an exponential decrease and reaches the value of around 2 mL/min/mmHg at the end of HD, similarly to the values reported in previous studies^5,11,12^. At the beginning of HD, Kr tends to infinity given the infinitesimally small changes in plasma oncotic pressure with respect to the initial value (see Eq. 2), which is why we show only the data from the 5th minute onwards for clarity.

**Figure 3:**
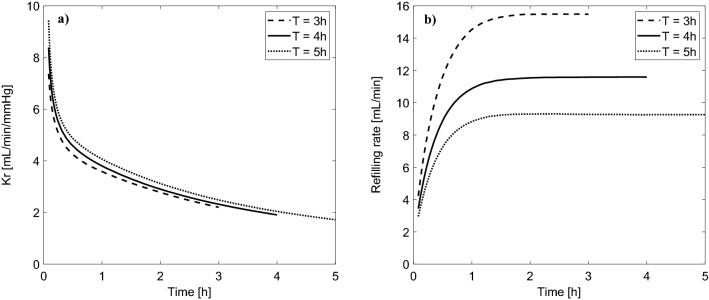
Simulated changes of (**a**) vascular refilling coefficient (Kr) and (**b**) vascular refilling rate (the sum of transcapillary fluid absorption and the total flow of lymph) during hemodialysis sessions of different duration (priming saline discarded) with the same total ultrafiltration of 3L and the whole-body capillary hydraulic conductivity (L_p_S) of 6 mL/min/mmHg.

Interestingly, for a shorter (3 h) or longer (5 h) session duration (with the same total ultrafiltration), the end-of-dialysis value of Kr is pretty similar (close to 2 mL/min/mmHg), despite the markedly different rates of vascular refilling (see Fig. 3). Moreover, changing the rate of ultrafiltration between 10 and 15 mL/min (corresponding to the total ultrafiltration between 2.4 and 3.6 L) had also a negligible effect on Kr during HD, despite the non-negligible difference in relative blood volume reduction (−6% vs −10%)—see Fig. S1 in the Supplementary material.

**Figure 4:**
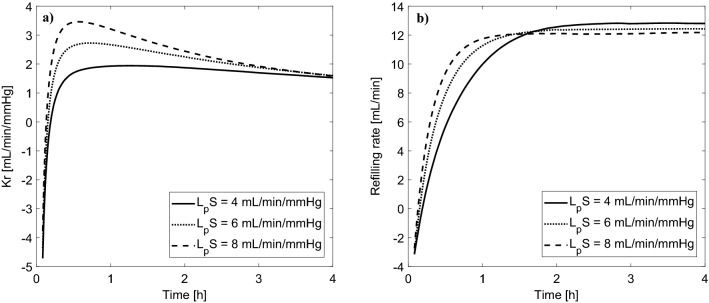
Simulated changes of (**a**) vascular refilling coefficient (Kr) and (**b**) vascular refilling rate (the sum of transcapillary fluid absorption and the total flow of lymph) during 4 h hemodialysis sessions with different values of the whole-body capillary hydraulic conductivity (L_p_S), with the priming saline infused to the patient.

In Fig. 4 we show the analogous changes of Kr and vascular refilling rate as in Fig. 2 but for the case with the priming saline infused to the patient. Again, for most of the dialysis duration, Kr decreases; however, in this case, at the beginning of dialysis Kr tends to minus infinity due to the fact that the infusion of priming saline leads to a transient increase in transcapillary filtration, which translates into a negative rate of vascular refilling that lasts for several minutes until the ultrafiltration causes a decrease in the net transcapillary fluid filtration. This phenomenon leads to a substantial change in the overall shape of the Kr curve in the early part of HD.

**Figure 5:**
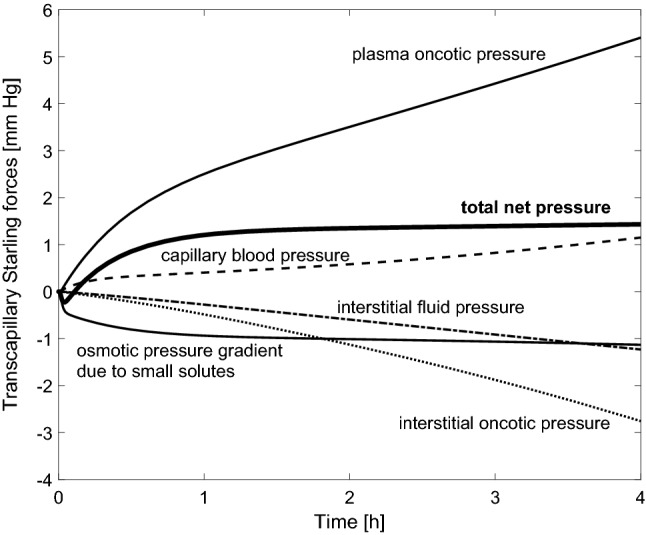
Simulated changes in the transcapillary Starling forces (hydraulic/hydrostatic and osmotic pressures acting across the capillary walls) during a standard 4 h hemodialysis session with the priming saline discarded. The rising curves indicate Starling forces acting in favor of transcapillary absorption, whereas the declining curves reflect Starling forces acting in favor of transcapillary filtration.

Apart from the initial phase of HD with the priming saline infused, it can be said, therefore, that Kr decreases during HD, even though L_p_S remains constant in our simulations. This can be explained by the fact that, contrary to the assumption by Tabei et al.^11,12^, during HD it is not only plasma oncotic pressure that changes but also other determinants of microvascular exchange, including interstitial oncotic pressure, capillary blood pressure, interstitial hydrostatic pressure as well as osmotic pressure exerted by small solutes (see Fig. 5). As a result, the total net transcapillary pressure (the sum of all hydraulic/hydrostatic and osmotic/oncotic pressures) that drives the intradialytic absorption of fluid from tissues increases much less than plasma oncotic pressure alone, as shown in Fig. 5. Note that the intradialytic pressure changes are shown in this figure as ‘forces’ driving the transcapillary absorption of fluid, e.g. a declining interstitial oncotic pressure curve reflects the increasing interstitial oncotic pressure that acts against transcapillary absorption.

As far as small solutes are concerned, the initial decrease of their osmotic pressure in plasma (see Fig. 5) is due to the intense removal of some solutes in the dialyzer (mainly urea and creatinine) that causes a small disequilibrium between their concentrations in plasma water and interstitial water. This concentration gradient of solutes being removed in the dialyzer stabilizes relatively quickly and is eventually compensated by solutes that are absorbed from the dialysis fluid (e.g. bicarbonate ions).

The overall transcapillary osmotic pressure gradient due to small solutes that is shown in Fig. 5 as slightly building up during HD (towards negative values) is mainly due to various anions (e.g. H_2_PO_4_^-^, HPO_4_^2-^, PO_4_^3-^, or SO_4_^2^ treated collectively as ‘other anions’ with the average charge −2) that in the model are assumed to travel across the capillary walls in a way that ensures electroneutrality of both plasma and interstitial fluid (the reduced capillary leakage of plasma proteins during HD leads to the shift of some of these ‘other anions’ from plasma to the interstitial fluid causing some extra osmotic pressure partly reducing the effective increase of plasma oncotic pressure).

In the basal scenario (priming saline discarded, L_p_S = 6 mL/min/mmHg), the lymph absorption of the interstitial fluid and its drainage to the veins decreased by circa 14% during HD (from 6.6 mL/min to 5.7 mL/min) due to the reduction of the interstitial fluid pressure (from 0.4 mmHg to −0.8 mmHg). However, when we divided the lymph flow by L_p_S to compare it with the Starling forces driving transcapillary vascular refilling (see the Supplementary material), its intradialytic decrease was only 0.15 mmHg, and hence it was negligible compared to the changes of hydraulic/hydrostatic and oncotic pressures of plasma and interstitial fluid.

**Figure 6:**
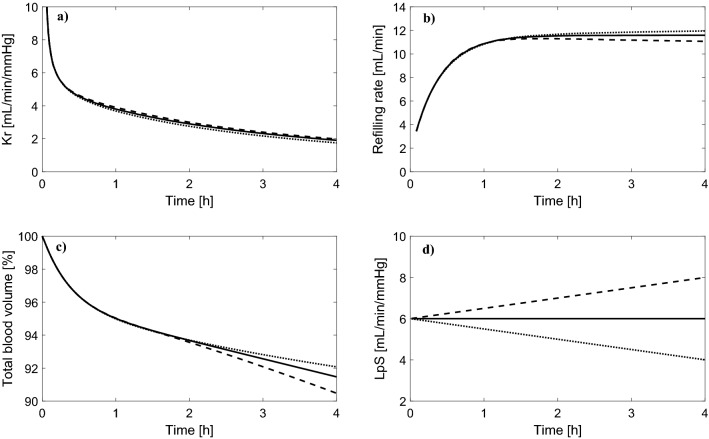
Simulated intradialytic changes in (**a**) vascular refilling coefficient (Kr), (**b**) vascular refilling rate, and (**c**) blood volume during a standard 4 h HD session (priming saline discarded) with the whole-body hydraulic conductivity (L_p_S) constant, decreasing, or increasing during HD, as shown in panel **d**).

Having shown that Kr decreases during HD even if L_p_S is constant, we then performed additional simulations to see how Kr would change during HD if L_p_S was actually decreasing, as hypothesized by Tabei et al.^11,12^, or if L_p_S was increasing during HD (less likely). As shown in Fig. 6, a gradual intradialytic increase or decrease in L_p_S by 33% had almost no impact on Kr (both in terms of intradialytic profile as well as the end-of-dialysis value). Paradoxically, by increasing L_p_S during our HD simulation we obtained slightly lower vascular refilling rates, which led eventually to a slightly higher decrease in blood volume at the end of HD (see Fig. 6 b and c). This can be explained by the fact that an increase of L_p_S in the model leads to an increase in the transcapillary flow through all types of capillary pores, including the large pores for which, even during HD, there is an ongoing filtration of fluid and proteins out of capillaries. This means that with a higher L_p_S there is more plasma proteins leaking through the large pores, which partly reduces the dialysis-induced increase in plasma oncotic pressure, thus reducing the transcapillary absorption of fluid that takes place through the small pores^25^.

**Figure 7:**
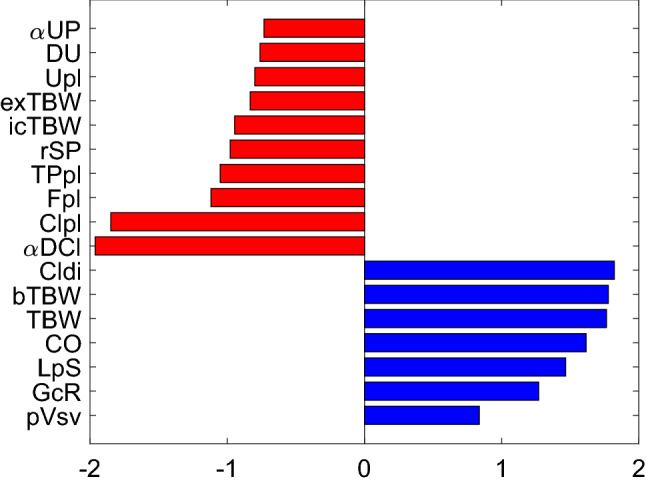
Relative sensitivity of the simulated Kr value at the 5th minute of HD (priming saline discarded) to the values of selected model parameters (only the parameters with the relative sensitivity above ± 0.5 are shown). Symbol meanings: αUP—fraction of LpS attributed to large pores, DU—urea clearance (used also for all other solutes in the model, except for creatinine and bicarbonate), Upl—pre-dialysis plasma urea level, exTBW and icTBW—fractions of normal total body water attributed to extravascular extracellular space and extravascular intracellular space, respectively, rSP—small pore radius, TP—pre-dialysis total protein level, Fpl—normal plasma water fraction, Clpl—pre-dialysis plasma chloride level, αDCl—Gibbs-Donnan coefficient for chloride across the dialyzer membrane, Cldi—chloride concentration in the dialysis fluid, bTWB—fraction of normal total body water attributed to blood, TBW—total body water, CO—normal cardiac output, LpS—whole-body capillary hydraulic conductivity, GcR—low-pressure (cardiopulmonary) gain of the baroreflex mechanism controlling vascular resistance, pVsv—normal fraction of total blood volume attributed to small veins and venules. For figure clarity, the following parameters (sensitivities) were not shown: Napl—pre-dialysis plasma sodium level (−8.7), Nadi—sodium concentration in the dialysis fluid (9.0), Fpl0—pre-dialysis plasma water fraction (12.4), αDNa—the Gibbs-Donnan coefficient for sodium across the dialyzer membrane (9.4).

Finally, given that in the early phase of HD L_p_S has the highest impact on Kr (see Fig. 2a), we analyzed the possibility of using Kr calculated during that phase to estimate L_p_S. For this purpose, we studied Kr at the 5th minute of HD (case with the priming saline discarded) and calculated its relative sensitivity to the values of all model parameters. Note that the relative sensitivity 1 means that a 1% change in a given parameter value would lead to a 1% change in Kr. In Fig. 7 we show the parameters to which Kr is most sensitive to (for the description of parameters, see the figure legend). L_p_S is, indeed, one of those parameters; however, there are 20 other parameters to which Kr has a similar or higher sensitivity. In particular, Kr is quite sensitive to the magnitude of chloride exchange in the dialyzer (governed by the concentration of chloride in the pre-dialysis plasma, Cl_pl_, and in the dialysis fluid, Cl_di_, as well as by the Gibbs-Donnan coefficient for chloride across the dialyzer membrane, αDCl). It is even more sensitive to sodium exchange in the dialyzer (again, governed by the level of sodium in the pre-dialysis plasma and in the dialysis fluid, the Gibbs-Donnan coefficient for sodium, as well as the pre-dialysis plasma water fraction—for figure clarity, the relative sensitivities of Kr to these parameters are listed in the figure legend). Some of these parameters are relatively easy to measure (e.g. solute concentrations in plasma or in the dialysis fluid); however, many of them are not possible to measure at all (e.g. the fraction of L_p_S attributed to ultrasmall pores, fraction of total blood volume attributed to small veins and venules, or the gain of the baroreflex mechanism controlling vascular resistance).

A similar sensitivity analysis but for the end-of-dialysis Kr value is shown in the Supplementary material (Fig. S2). In that case, Kr shows much lower sensitivity to all model parameters (including those related to sodium exchange in the dialyzer) with the relative sensitivity to L_p_S of only −0.01.

## Discussion

At first glance, the refilling coefficient Kr, as proposed by Tabei et al.^11,12^ and given by Eq. (3), may seem as a useful marker of the whole-body capillary hydraulic conductivity (L_p_S) in dialysis patients with a potential use in the assessment of patient’s fluid status or estimation of the dry weight^14,15^. However, their definition of Kr assumes that plasma oncotic pressure (π_pl_) is effectively the only variable Starling force during HD, while other parameters potentially influencing Kr—including the hydrostatic pressure gradient between capillary blood and interstitial fluid (ΔP = P_c_—P_is_), interstitial oncotic pressure (π_is_), and lymph flow (L)—are assumed to be constant. In the present study, we challenged these assumptions and hypothesized that in real dialysis conditions (when all of the above-mentioned factors may be subject to changes), Kr may actually not be an accurate marker of L_p_S. Our model-based simulations confirm this hypothesis.

### Changes in the Starling forces during HD

On the one hand, we showed that ΔP can, indeed, be assumed as almost constant during HD (given that the decrease in the capillary blood pressure that promotes vascular refilling is estimated to be almost the same as the decrease in the interstitial hydrostatic pressure that acts in the opposite way, as shown in Fig. 5). Our simulations also showed that, even though the total lymph flow is subject to a marked reduction during HD, when expressed in the units of Starling forces (mm Hg), its intradialytic changes are very small compared to the main (classic) Starling forces and can be hence neglected without affecting the assessment of Kr. On the other hand, as shown in Fig. 5, the dialysis-induced changes in the interstitial oncotic pressure should not be neglected. In fact, they are expected to be of the same order of magnitude as the changes in the plasma oncotic pressure and, when accounted for, the net increase in the transcapillary pressure gradient that drives vascular refilling is much smaller compared to the increase in plasma oncotic pressure alone. As a result, even if L_p_S remains constant (as in our basal simulations), Kr decreases during HD in an exponential-like manner, which is due to the overestimation of the effective net Starling force driving vascular refilling. Therefore, the decrease of Kr observed by Tabei et al.^11^ and Iimura et al.^12^, contrary to their hypothesis, does not imply a dialysis-induced reduction in L_p_S, since such a decrease is an entirely expected behavior of Kr related to the aforementioned assumptions.

### Possible changes in LpS during HD

Iimura et al. hypothesized that the reduction of Kr during HD may be caused by a dialysis-induced decrease of plasma ANP that they observed in their study^12^, with likely limited impact of plasma noradrenaline^38^. This was a plausible hypothesis as it is well known that plasma ANP is elevated before dialysis and decreases during HD^39,40^ (due to its suppressed secretion following the reduction of atrial pressure as well as due to its clearance in the dialyzer^41^) and that ANP raises microvascular permeability, as has been shown in both animals^42,43^ and humans^44^. Moreover, Schneditz et al.^2^ and Yashiro et al.^15,45^ showed that the whole-body capillary filtration coefficient (estimated similarly to Kr) is positively correlated with the level of overhydration, which suggested that a decrease in L_p_S during HD may be related to a progressing reduction in overhydration (likely associated with a drop in ANP). However, our simulations indicate that, even if L_p_S was actually decreasing during HD (for whatever reason), it should not affect Kr, as shown in Fig. 6.

### Previous studies

It was shown previously by Pietribiasi et al. that the decrease in Kr observed during HD could be largely explained by the assumed lack of intradialytic changes in the interstitial Starling forces and the flow of lymph (except for patients with the highest initial Kr)^46^. They also showed that those assumptions could be valid only if the capillary blood pressure (assumed constant) decreased during dialysis approximately three times more than the interstitial fluid pressure^46^, which is rather unlikely given the autoregulatory capacity of the capillary beds^24^. The present study provides a more complete mathematical analysis of the vascular refilling process, given that in our model the capillary blood pressure is a variable that depends on the state of the whole cardiovascular system (particularly the venous system), as opposed to a constant value assumed in the model with one plasma compartment. Moreover, compared to the model by Pietribiasi et al., the model used in the present study accounts for the two most important plasma protein fractions (albumin and globulins) and their different behavior in terms of transcapillary leakage and refilling, thus providing a likely better estimation of plasma and interstitial oncotic pressures. Furthermore, in the description of fluid transport across the capillary walls we included the impact of osmotic pressure of small solutes (the last term in Eq. 4). Even though the concentrations of ions and small molecules on the two sides of the capillary wall are normally equilibrated due to their high permeability and a very low reflection coefficient, transient transcapillary concentration gradients may occur during dialysis (especially for the solutes being removed in the dialyzer, such as urea or creatinine).

A few years before the refilling coefficient was defined by Tabei et al.^11^, Schneditz et al. used a similar approach to calculate L_p_S (which they called simply L_p_)^2^. They considered a three-phase experimental procedure during the first hour of HD: (1) 20 min of blood volume equilibration with pure dialysis and no ultrafiltration, (2) 20 min of intense ultrafiltration (equivalent to 1 h of scheduled ultrafiltration), and (3) 20 min of blood volume recovery with no ultrafiltration. They found no significant difference between L_p_ calculated for the ultrafiltration phase and the recovery phase, although there were some differences in individual patients. More importantly, however, Schneditz and colleagues were able to fit the relative blood volume (RBV) curves from both ultrafiltration and recovery phases using model-based simulations with a constant (fitted) value of L_p_ and accounting for the changes of the interstitial oncotic pressure. If the latter were not included in their model, they most probably would not be able to fit the RBV curves using a constant L_p_ value, since in such a case L_p_ would need to change during HD just like Kr in the study by Tabei et al. or in our simulations.

Schneditz et al.^2^ proposed that the changes in blood volume observed over a short period of ultrafiltration during the initial phase of HD could be used in a model-based approach to estimate the (assumingly constant) whole-body filtration coefficient (L_p_S), which could be then used to determine the safe ultrafiltration rate for the remaining of the dialysis session. We agree that this could be theoretically possible; however, this would need to be done during the very early phase of HD, when the refilling rate is most sensitive to L_p_S (see Fig. 2), which may be problematic given that during this phase body fluids are typically not in a steady state^47^, and hence the observed blood volume changes may not always enable an accurate fitting of the model (note that Schneditz et al. started all their experiments with a 20-min equilibration phase). Moreover, as shown by our sensitivity analysis, even though L_p_S clearly affects the refilling process (and the value of Kr) at the early phase of HD, it cannot be accurately estimated from Kr alone, given the interference from several other, often unmeasurable, parameters (see Fig. 7).

### Revised starling principle

As mentioned in the Introduction, one of the limitations of the present study is that in our model we used the classic Starling principle of microvascular fluid exchange, thus ignoring the possible differences in terms of oncotic and hydrostatic pressure between the bulk interstitial fluid and the sub-glycocalyx fluid, i.e. the fluid between the abluminal side of glycocalyx and the tight junction strand within the inter-endothelial clefts in continuous (non-fenestrated) capillaries, as advocated by the revised or extended Starling principle^10,17,48^. The classic approach allowed us to refer directly to the discussed works by Tabei et al. as well as to other studies mentioned above, all of which employed the classic Starling principle. In order to reflect the extended Starling principle, our model would need to be substantially extended by either: (1) adding a sub-glycocalyx compartment (or possibly more sub-compartments of the interstitial fluid, as proposed by Curry and Michel^21^) with the description of convection and diffusion of macromolecules between that compartment and the interstitial fluid, which would depend on the flow rate (velocity) of the filtration flow through the orifices in the junction strand, or (2) employing a spatially distributed model of pressure and protein concentration fields behind the glycocalyx, as done by Hu and Weinbaum^28^, or (3) modelling the capillary wall as a two-membrane system (glycocalyx + endothelium), as done by Facchini et al^49,50^. Any of the above approaches would increase substantially the level of complexity of our already relatively complex model, but, more importantly, as outlined below, we believe that the possible error introduced by using the classic approach should not affect our conclusions with respect to the deficiencies of Kr.

Firstly, as shown in the simulations by Hu and Weinbaum^28^ and the experiments by Adamson et al.^51^, a large difference in the oncotic pressure between the sub-glycocalyx fluid and the interstitial fluid is observed only at very high filtration rates, when filtration of the macromolecule-deficient fluid through the inter-endothelial clefts washes out the macromolecules from the space behind the glycocalyx, while the high velocity of flow through the orifices in the junction strand precludes them from diffusing upstream from the interstitial fluid (assuming that there is an alternative trans-endothelial pathway for the macromolecule transport to the interstitial fluid, i.e. the large pores, as used in our model, which may represent either the vesicular transport or larger gaps in endothelium and glycocalyx^20,28^). In this case, the oncotic pressure of the sub-glycocalyx fluid is very low (even close to zero at very high filtration rates), and hence the trans-glycocalyx oncotic pressure gradient opposing filtration is very high. So, at the beginning of HD, when there is still filtration through the inter-endothelial clefts (small pores), using the interstitial instead of sub-glycocalyx oncotic pressure in the Starling equation may underestimate the oncotic pressure gradient and hence overestimate filtration through the small pores. In our model, this would automatically mean an underestimation of filtration through large and ultrasmall pores, given that we define the initial steady-state conditions in such a way as to obtain the overall filtration rate equal to the assumed rate of lymph absorption (for this we adjust the initial mean capillary blood pressure). Also, just like the oncotic pressure behind the glycocalyx may be different (lower) from the oncotic pressure of the interstitial fluid, the sub-glycocalyx hydrostatic/hydraulic pressure may be different (higher) from the hydrostatic pressure of the interstitial fluid (again, this is a phenomenon observed mainly at high filtration rates^28^). Overall, the profile of the oncotic and hydrostatic/hydraulic pressure on the abluminal side of glycocalyx is such that the actual filtration takes place only around the orifices in the junction strand, whereas in the other parts of the clefts the flow rate is zero or near zero^28^. When the filtration rate is low or close to normal, the phenomena described above are either not observed^52^ or much less conspicuous with the sub-glycocalyx oncotic pressure reaching almost 90% of the oncotic pressure in the bulk interstitial fluid^28,51^.

When the flow direction in the inter-endothelial clefts changes to the opposite, i.e. when fluid is absorbed from tissues, as for the majority of the modelled dialysis sessions (mainly due to increase in plasma oncotic pressure and partly due to decrease in capillary blood pressure), the oncotic pressure of the sub-glycocalyx fluid is no longer lower than that of the interstitial fluid; in fact, it is even higher due to reflection of macromolecules from the glycocalyx layer and their accumulation in the sub-glycocalyx space^21^ (the magnitude of this effect would depend on the rate of fluid absorption and the velocity of fluid through the junction strand openings that would affect the diffusion of macromolecules back to the interstitial fluid). In most tissues with continuous (non-fenestrated) endothelia, such as skeletal muscles and skin, such absorption of fluid from the tissue following a reduction in capillary blood pressure or increase in plasma oncotic pressure is possible only transiently until a new steady state is established across the glycocalyx (a state of filtration, as indicated by the Michel-Weinbaum model^22,23,53^ and as shown experimentally, albeit only in frog and rat microvessels^51,53^)—this takes usually 15–30 minutes^10,48^ but may continue for more than an hour^17,21^. During HD, however, there is no step-like change in the conditions for microvascular exchange, as typically considered in the studies devoted to the extended Starling principle, nor a more gradual but still relatively quick change of microvascular conditions as in hemorrhage or following an infusion in fluid therapy^54,55^. Instead, during a typical HD session there is a progressing (lasting 3–5 h) increase in the plasma oncotic pressure (mainly due to ultrafiltration in the dialyzer, and partly due to reduced transcapillary protein leakage) as well as a less prominent but also progressing decrease in the capillary blood pressure, both of which preclude the system from reaching a steady state and provide a continuous drive for fluid absorption, as shown in several studies^2–4,56,57^.

In our model, the rate of capillary fluid absorption may be somewhat overestimated by using the interstitial instead of sub-glycocalyx oncotic pressure, but, given the associated overestimation of the initial rate of filtration through the small pores (as described above), it is unlikely that these overestimations are significant on the whole-body level. As already mentioned, our overestimation of initial filtration rate through the small pores translates into underestimation of filtration through the large pores. If the initial filtration through the large pores was in fact higher, it would have been also higher for the whole dialysis session (becoming only slightly reduced due to decreasing capillary blood pressure), and hence the transcapillary absorption of fluid would need to be even larger than in our model to provide sufficient vascular refilling (note that the latter could not be achieved through the lymphatic system alone, given that the assumed flow of lymph was 8 L/day in normal conditions, raised to around 9.6 L/day in the pre-dialysis state of fluid overload, whereas the ultrafiltration in the dialyzer was 3 L over 4 h; it would not be possible even if capillary filtration ceased during HD, which is not the case for the large pore pathway^25,26^). Moreover, the glycocalyx-related phenomena are much less conspicuous in fenestrated capillaries^58,59^, and hence they would be somewhat less important on the whole-body level.

Overall, even if the Michel-Weinbaum model was appropriate for the assessment of the whole-body microvascular fluid exchange in humans (which is doubted by some researchers^60,61^), we believe that the possible inaccuracies resulting from ignoring the differences between the sub-glycocalyx fluid and the interstitial fluid in the equation describing the fluid exchange through the small pores (endothelial clefts and glycocalyx) would not invalidate our general observations with regard to Kr.

### Blood priming procedure and steady-state conditions

Tabei et al. calculated Kr at each hour of HD and then extrapolated the results to the beginning of HD using exponential curves^11^. As shown by our simulations, this would have been a correct approach in the case with the priming fluid discarded at the beginning of the HD procedure (see Fig. 2). However, when the priming fluid is not discarded but infused to the patient (as done in the experiments by Tabei et al.), the overall shape of the Kr curve is markedly different in the early phase of HD, when Kr becomes transiently negative (see Fig. 4). Moreover, regardless of the priming procedure, after filling of the extracorporeal circuit with the patient’s blood, the body fluids are not in a steady state, which further distorts the calculation of Kr, which assumes that at time point 0 there is no vascular refilling. Tabei et al. waited 15 min after filling the extracorporeal circuit with blood in an attempt to obtain a steady state before starting ultrafiltration. However, according to our simulations, one would need around 1 h to reach steady-state conditions after the infusion of the priming saline (or at least 45 min to come close to the steady state).

### Estimation of plasma volume changes

Another limitation of Kr is that to calculate it one needs to know the instantaneous rate of plasma volume changes in absolute terms. While this rate can be easily calculated within a mathematical model framework, obtaining real-world clinical data would require an accurate estimation of initial (pre-dialysis) plasma volume and reliable tracking of its subsequent relative changes. To this end, several approaches have been proposed. Schneditz et al. calculated the initial plasma volume from the pre-dialysis hematocrit and blood volume estimated from the lean body mass, and then used blood and plasma density measurements to calculate the relative plasma volume changes during HD^2^. They analysed a relatively short period of time (20 min) for which they assumed a constant rate of plasma volume changes. Tabei et al.^11^, on the other hand, calculated the initial blood volume from the total body weight and used hematocrit measurements to estimate relative plasma volume changes during HD to which they fitted an exponential curve, the derivative of which was used as the instantaneous rate of plasma volume changes (as done also by Pietribiasi et al.^5^). Both approaches are, however, not ideal—the first one neglecting the short-term differences in the rate of plasma volume changes (particularly high in the early phase of HD, when Kr should be estimated), and the second one requiring longer periods of measurements to derive an analytical form of the plasma volume curve (fitted to data). Moreover, the second approach assumes that the total volume of erythrocytes remains constant during HD, which may not necessarily be true, particularly with hyper- or hyponatremic dialysis fluid^62,63^. As for the estimation of the initial absolute blood volume, instead of using anthropometric formulae (likely inaccurate in fluid overloaded dialysis patients, as shown by Mitra et al. using indocyanine green^64^), one may use the blood dilution technique proposed more recently by Schneditz and Kron^65,66^, although this is again not unambiguous when done at the beginning of HD^67^.

### Protein transport

Furthermore, the refilling coefficient defined by Tabei et al.^11^ assumes that proteins cannot pass through the capillary walls (σ = 1), which leads to overestimation of the effective plasma oncotic pressure in Eqs. (1) and (4). Moreover, HD may cause not only a change in the total concentration of plasma proteins but also may affect the composition of plasma proteins due to protein leakage through large capillary pores and protein refilling through small pores^25^. As a result, plasma oncotic pressure calculated from the total plasma proteins may lead to a further bias in the estimation of L_p_S by Kr. Therefore, when calculating the changes in plasma and interstitial oncotic pressures during HD one should track not the total protein content but various protein fractions, at least differentiating between albumin and non-albumin proteins that can have largely different reflection coefficients and permeability through the capillary walls, thus affecting the changes of the oncotic pressure.

### Whole-body aggregation

A more general limitation of Kr is that it is meant to represent the whole-body refilling coefficient as a substitute of the aggregated hydraulic conductivity of all capillaries in the body (L_p_S). Capillaries in various tissues may, however, show different properties in terms of water permeability, which may change during HD to various extent, thus affecting the average value. Moreover, during HD the pattern of blood flow distribution among different microvascular beds may change, thus further affecting the aggregated L_p_S^2﻿^, which would then change not because of changes in the circulating hormones or fluid status but simply because of an altered blood flow distribution. Note that such a change in blood flow distribution could affect Kr not only through a direct change in L_p_S but also through a potential change in the aggregated protein reflection coefficient.

## Summary

In conclusion, given the substantial distortion of Kr as a marker of the whole-body capillary filtration coefficient (due to the associated assumptions and the potential influence of other parameters) as well as the technical difficulties associated with a correct calculation of Kr from a mathematical point of view, we suggest not to use Kr as a marker of vascular refilling or patient’s fluid status.

## Supplementary Information

Supplementary Information.

## Data Availability

The detailed description of the mathematical model used in this study as well as the values of all model parameters and initial conditions may be found in our previous work, as mentioned in the manuscript.
