# Supplementary material for: Vascular refilling coefficient is not a good marker of whole-body capillary hydraulic conductivity in hemodialysis patients: insights from a simulation study
Source: Sci Rep. 2022 Sep 10;12:15277. doi: 10.1038/s41598-022-16826-8 (PMC9464211; doi:10.1038/s41598-022-16826-8)
Supplement: Supplementary file 1 — Supplementary Information. [file 41598_2022_16826_MOESM1_ESM.pdf]

Leszek Pstras, Jacek Waniewski, Bengt Lindholm
“Vascular refilling coefficient is not a good marker of whole-body capillary hydraulic conductivity in hemodialysis patients: insights from a simulation study”

SUPPLEMENTARY MATERIAL

Refilling coefficient formula

From equation (2) we have:

$$-J_v(t) + L(t) = \frac{dV_p}{dt} + UF(t) \qquad (12)$$

Hence, using equation (1):

$$-K_f\left[\left[P_c(t) - P_{is}(t)\right] - \sigma\left[\pi_{pl}(t) - \pi_{is}(t)\right]\right] + L(t) = \frac{dV_p}{dt} + UF(t) \qquad (13)$$

Assuming that: 1) the hydrostatic pressure difference between the capillary blood and interstitial fluid $\Delta P = P_c(t) - P_{is}(t)$ is constant during dialysis, 2) the interstitial oncotic pressure (π_{is}) and lymph flow (L) do not change during dialysis, 3) the protein reflection coefficient (σ) is equal to 1, and 4) ultrafiltration (UF) is constant, we have:

$$-K_f\left[\Delta P - \left[\pi_{pl}(t) - \pi_{is}(0)\right] - \frac{L(0)}{K_f}\right] = \frac{dV_p}{dt} + UF \qquad (14)$$

From the initial steady state conditions:

$$\frac{L(0)}{K_f} = \Delta P - \left[\pi_{pl}(0) - \pi_{is}(0)\right] \qquad (15)$$

Using equation (15) in (13) we get:

$$-K_f\left[-\left[\pi_{pl}(t) - \pi_{pl}(0)\right]\right] = \frac{dV_p}{dt} + UF \qquad (16)$$

And finally, expressing K_f as a time-dependent refilling coefficient Kr:

$$Kr(t) = \frac{\frac{dV_p}{dt} + UF}{\pi_{pl}(t) - \pi_{pl}(0)} \qquad (17)$$

Note that in the original formulation of the refilling coefficient Tabei et al.[1,2] did not consider lymph flow as part of the vascular refilling, and hence in their derivation of Kr there is no mention of L(0) or L(t), both of which are therefore inexplicitly assumed to be equal to 0, implying that under steady-state conditions there is no net filtration out of the capillaries. This is, however, equivalent to the assumption that the lymph flow is constant during HD, as done in our derivation above (as long as K_f is also constant). In their equations, they also did not consider the protein reflection coefficient (σ), which is equivalent to assuming that σ is equal to 1, as done here.

References

1 Tabei, K., Nagashima, H., Imura, O., Sakurai, T. & Asano, Y. An index of plasma refilling in hemodialysis patients. *Nephron* **74**, 266-274, doi:10.1159/000189320 (1996).

2 Iimura, O., Tabei, K., Nagashima, H. & Asano, Y. A study on regulating factors of plasma refilling during hemodialysis. *Nephron* **74**, 19-25, doi:10.1159/000189276 (1996).

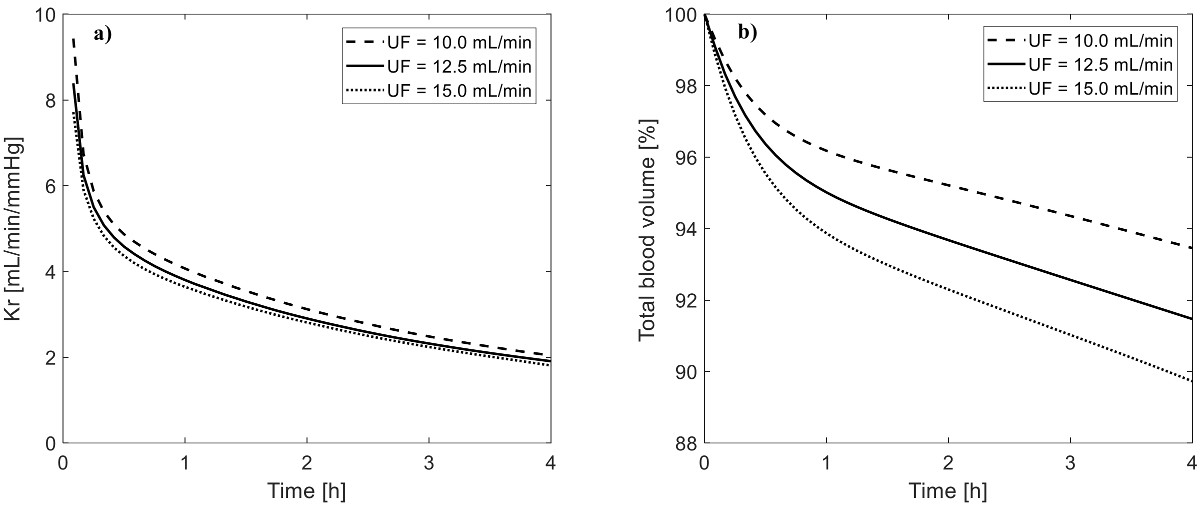

Fig. S1. Simulated intradialytic changes in **a)** vascular refilling coefficient (Kr), and **b)** intravascular blood volume during a standard 4h hemodialysis session (basal case with priming saline discarded) for different rates of ultrafiltration (with the same patient fluid overload of 3L).

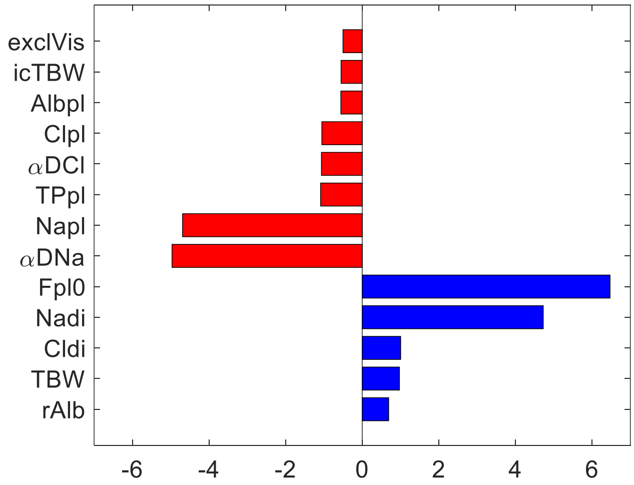

Fig. S2. Relative sensitivity of the simulated end-of-dialysis Kr value (case with the priming saline discarded) to the values of selected model parameters (only the parameters with the relative sensitivity above ±0.5 are shown). Symbol meanings: exclVis – fraction of the interstitial space excluded to proteins, icTBW – fractions of normal total body water attributed to extravascular extracellular space and extravascular intracellular space, respectively, Albpl – pre-dialysis level of plasma albumin, Clpl – pre-dialysis plasma chloride level, αDCl – the Gibbs-Donnan coefficient for sodium across the dialyzer membrane, TPpl – pre-dialysis total protein level, Napl – pre-dialysis plasma sodium level, αDNa – the Gibbs-Donnan coefficient for sodium across the dialyzer membrane, Fpl0 – pre-dialysis plasma water fraction, Nadi – sodium concentration in the dialysis fluid, Cldi – chloride concentration in the dialysis fluid, TBW – total body water, rAlb – radius of albumin.
